# Preoperative evaluation of pulmonary hypertension in lung transplant candidates: echocardiography versus right heart catheterization

**DOI:** 10.1186/s12872-022-02495-y

**Published:** 2022-02-16

**Authors:** Tal Abu, Amos Levi, David Hasdai, Mordechai R. Kramer, Tamir Bental, Tali Bdolah-Abram, Arthur Shyovich, Abed Samara, Hana Vaknin-Assa, Leor Perl, Dror Rosengarten, Yaron Shapira, Ran Kornowski, Keren Skalsky

**Affiliations:** 1 Faculty of Medicine, The Hebrew University of Jerusalem, Jerusalem, Israel; 2 Department of Cardiology, Rabin Medical Center – Beilinson Hospital, 39 Jabotinsky St., 4941492 Petach Tikva, Israel; 3 Rabin Medical Center, Pulmonary Institute, Petach-Tikva, Israel; 4 Affiliated to Sackler Faculty of Medicine, Tel Aviv University, Tel Aviv, Israel

**Keywords:** Lung transplant, Right heart catheterization, Pulmonary hypertension

## Abstract

**Background:**

Right heart catheterization (RHC) and echocardiography are both routinely used for pulmonary artery systolic pressure (PASP) assessment in lung transplantation (LT) candidates, although this is not mandated by current guidelines. We aimed to explore the performance of echocardiographic PASP as an indicator of pulmonary hypertension in LT candidates, in order to assess the necessity of RHC.

**Methods:**

From a retrospective registry of 393 LT candidates undergoing RHC and echocardiography during 2015–2019, patients were assessed for the presence of pulmonary hypertension (PH), defined as mean pulmonary artery pressure (mPAP) above 20 mmHg, according to two methods—echocardiography and RHC. The primary outcome was the correlation between the PASP estimated by echocardiography to that measured by RHC. Secondary outcomes were the prediction value of the echocardiographic evaluation and its accuracy.

**Results:**

The mean value of PASP estimated by echocardiography was 49.5 ± 20.0 mmHg, compared to 42.5 ± 18.0 mmHg measured by RHC. The correlation between the two measurements was moderate (Pearson’s correlation: r = 0.609, *p* < 0.01). Echocardiography PASP measurements were moderately discriminative to diagnose PH, with an area under the curve (AUC) of 0.72 (95% CI 0.66–0.77). Echocardiographic overestimation of PASP of more than 10 mmHg was found in 35.0% of the patients, and underestimation was found in 11.6% of the patients.

**Conclusion:**

In the pre-surgical evaluation of LT candidates, echocardiographic estimation of PASP had moderate correlation and limited accuracy compared to the PASP measured by RHC. We thus recommend performing routine RHC to all LT candidates, regardless of the echocardiographic estimation of PASP.

**Supplementary Information:**

The online version contains supplementary material available at 10.1186/s12872-022-02495-y.

## Introduction

Lung transplant (LT) is a life-saving procedure for advanced stage of lung disease [1]. Pulmonary hypertension (PH), defined as mean pulmonary artery pressure (mPAP) above 20 mmHg [2], is common in those patients and is important to recognize prior to transplant. Its diagnosis gives priority on the waiting list for lung transplantation according to Lung Allocation Score [3] and may lead to initiation of specific drug therapy, aiming to lower it before the transplant operation [4, 5]. Moreover, elevated pulmonary pressure is a predictor of early death post transplantation [6–8].

The measurement of pulmonary artery systolic pressure (PASP) is the clinically relevant parameter since it is used in the pre-transplant lung allocation score. The latter aims to direct organs towards the 'right' patient on the waiting list based on a balance of short-term mortality and post-transplant survival, and it’s use has been to proven to decrease the number of deaths of patients on the wait list [3, 9, 10].

Right heart catheterization (RHC) is the gold standard for measuring pulmonary artery systolic pressure (PASP), mean pulmonary artery pressure (mPAP) and other cardiac hemodynamic parameters [5, 11, 12]. When performed in an experienced center, RHC is associated with 1.1% morbidity and 0.055% mortality complication rate [13]. Echocardiography is an acceptable non-invasive alternative for measuring PASP. It provides an estimation of PASP, by adding the regurgitant flow on continuous-wave Doppler over the tricuspid valve to the estimated right atrial pressure (RAP) [11, 12, 14, 15].

Whether non-invasive echocardiography is accurate enough to forego RHC and its complications in LT candidates is debatable. The Pulmonary Council of the International Society for Heart and Lung Transplantation (ISHLT) consensus document on LT candidates published in 2015 states that echocardiography is recommended in all LT candidates in their preoperative evaluation [16]. The ESC guidelines for the diagnosis and treatment of PH states that an echocardiography should be used as a screening tool for PH in all patients with suspected elevated pulmonary pressure, but when treatment of PH is being considered, echocardiography alone is not sufficient to support a treatment decision, and RHC is required [5]. In practice, both tests, echocardiography and RHC, are routinely used in order to estimate and measure PASP of LT candidates in many centers worldwide. We aimed herein to examine the We aimed to explore the performance of echocardiographic PASP as an indicator of pulmonary hypertension in a large contemporary cohort of LT candidates.

## Methods

This observational study is based on a retrospectively cohort of 461 LT candidates evaluated for transplant by RHC at two campuses of Rabin Medical Center from January 2015 through December 2019. Of these, we analyzed 393 patients who underwent echocardiography within one year from RHC. For each LT candidate, we collected additional data about demographics (age and gender), medical history (e.g., Diabetic mellitus—DM, Hypertension—HTN, smoking, COPD, Chronic kidney disease—CKD, Coronary artery disease—CAD, Hyperlipidemia, Body Mass Index—BMI and lung failure etiology. Full echocardiographic assessment including left ventricle (LV) systolic function, right ventricular function and valvular disease were recorded. Right atrial pressure was assessed according to the inferior vena cava (IVC) size and collapsibility (> 2.1 cm, collapse < 50%: RAP 15 mm Hg) [17]. PASP was assessed by adding the trans tricuspid pressure gradient during systole (estimated by applying the Bernoulli equation on the measured maximal tricuspid regurgitant jet velocity with continuous wave doppler) to the estimated right atrial pressure [18–20]. RHC was performed via the venous femoral or cubital fossa access using a 6–7 FR Swan Ganz catheter. Direct hemodynamic measurements of the right sided chambers as well as the pulmonary artery pressure were performed. Pulmonary capillary wedge pressure (PCWP) was measured by wedging the swan ganz catheter with an inflated balloon into a small pulmonary arterial branch. Cardiac output was calculated according to the Fick principle, using the arterial-venous oxygen content difference and the blood hemoglobin level.

The study protocol and data collection was approved by Rabin Medical Center's human research committee, according to the ethical guidelines of the 1975 declaration of Helsinki. Patients or the public were not involved in the design, or conduct, or reporting, or dissemination plans of the study.

The primary endpoint of the study was the diagnosis of PH in LT candidates, and the correlation between PASP estimated by echocardiography and PASP measured by RHC. Secondary endpoints were the prediction value of the echocardiographic assessment of PH and its accuracy. To examine the relationship between two quantitative variables, the Pearson correlation coefficient and the equation of the regression line were calculated. Comparison of quantitative variables was performed using the two-sample t-test for normally distributed variables or the Wilcoxon test for non-normally distributed variables. The association between two categorical variables was tested using either the Chi-Square test or the Fisher’s exact test as appropriate. ROC curves were plotted to study the diagnostic performance of echocardiography based PASP to diagnose PH (defined by RHC mPAP > 20 mmHg) and severely elevated pulmonary pressure (defined as RHC mPAP > 35 mmHg) [1]. For each ROC curve, maximal Youden’s index was calculated to define the optimal cut-off point to distinguish between patients with or without PH. The Bland–Altman method was used to plot the difference in PASP measurements for each patient (RHC PASP measurement minus estimated PASP per echocardiogram) against the mean of the two measurements. Statistical analysis was performed using IBM SPSS statistics software (version 25). A *p* value ≤ 0.05 was considered statistically significant for all analyses.

## Results

TR jet, allowing estimation of the PASP during echocardiography, was recognized in 351 out of 393 LT candidates undergoing RHC (89.1%). Patients were predominantly males (63.6%) with a mean age of 61.5 ± 8.3 years (Table 1). ILD was the most common primary lung disease in more than half of the patients, and COPD was the next most common diagnosis (Additional file 1: Lung transplant etiologies). Among these patients, approximately a third had diabetes mellitus and hypertension, more than a half were current or former smokers and almost a half were overweight or obese. History of CAD was documented in approximately a quarter of the patients (Table 1). The great majority of patients had good LV systolic function (ejection fraction (EF) > 55%) as estimated by echocardiography. Heart failure with reduced EF (≤ 55%) was recorded in a minority of patients, with only 1.6% patients with EF < 40%. 46 candidates (11.7%) had RV dysfunction (Table 2). The average PASP estimated by echocardiography was 49.5 ± 20.0 mmHg.

**Table 1:** Baseline characteristics of patients

	All patients N = 393
Age, mean (years)	61.5 ± 8.3
Male gender (%)	63.6
Diabetes (%)	35.4
Hypertension (%)	35.1
Hyperlipidemia (%)	36.9
Chronic kidney disease (%)	7.1
Current smoking (%)	37.9
Past smoking (%)	18.3
Steroids (%)	40.5
BMI, mean	26.2 ± 5.6
BMI categories (%)	
Underweight BMI ≤ 18	3.8
Normal 18 < BMI ≤ 25	27.2
Overweight 25 < BMI < 30	20.6
Obese BMI ≥ 30	21.6
CAD history (%)	23.9

BMI, Body Mass Index; CAD, chronic artery disease

**Table 2:** Right heart catheterization and echocardiographic data

	RHC	Echocardiography	*P* value
Estimated EF, mean (%)	Not recorded	59.1 ± 5.3	
RV dysfunction (%)		11.7	
Measurable TR (%)		89.1	
RA pressure, mean (mmHg)	5.1 ± 4.6	6.0 ± 3.2	0.89
PAP, mean (mmHg)	25.9 ± 11.5	Not recorded	
PA sys, mean (mmHg)	42.5 ± 18.0	49.5 ± 20.0	< 0.001
PCWP, mean (mmHg)	9.8 ± 6.1	Not recorded	
PVR, mean (mmHg)	4.2 ± 3.5		
CO, mean (L/min)	4.4 ± 1.4		
CI, mean (L/min/m^2^)	2.5 ± 0.8		

CO, cardiac output; CI, Cardiac Index; EF, ejection fraction; PA, pulmonary artery; PAP, pulmonary arterial pressure; PCWP, pulmonary capillary wedge pressure; PVR, pulmonary vascular resistance; TR, tricuspid regurgitation; RA, right atrium; RHC, right heart catheterization; RV, right ventricle

Pulmonary hypertension (mPAP > 20 mmHg) as measured by RHC was recorded in 251 patients (63.9%). The mean value of mPAP measured by RHC was 25.9 ± 11.5 mmHg and mean PASP was 42.5 ± 18.0 mmHg. Pulmonary vascular resistance (PVR) was elevated, with a mean value of 4.2 ± 3.5 Wood units. Mean PCWP was not elevated and the average cardiac output (CO) and cardiac index (CI) were both within the normal range (Table 2). Patients who had elevated pulmonary pressure were younger with a mean age 59.7 years versus 62.2 years, *p* = 0.021, and tended to have RV dysfunction (28.7%, *p* < 0.001). No other variables were found to be associated with elevated pulmonary pressure. The mean time interval between the echocardiography and RHC was 25 ± 372 days, and 78.3% of the patients have completed both tests within 90 days.

**Fig. 1:**
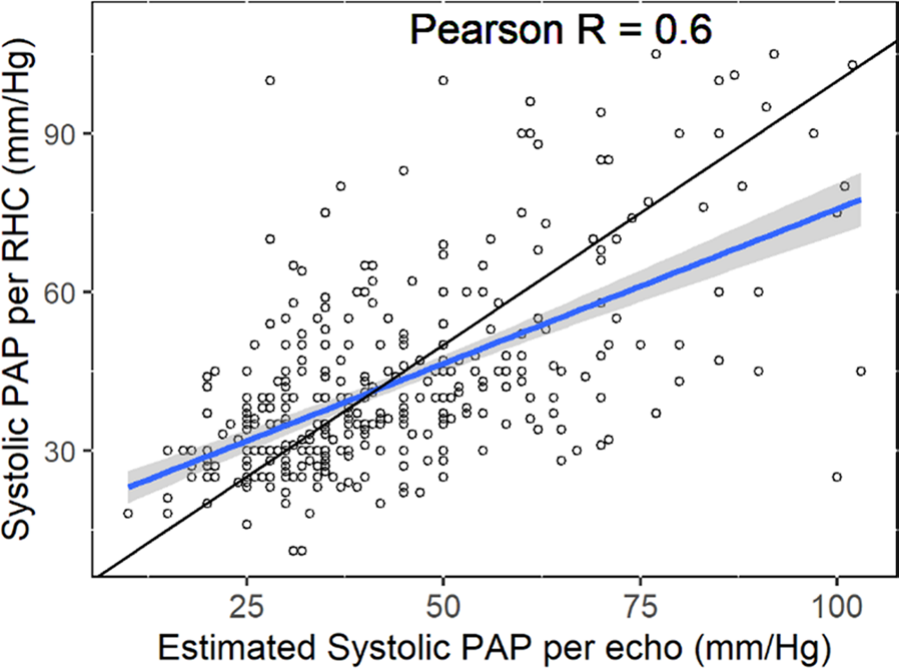
A scatter plot of the correlation coefficient (r = 0.6) between the PASP measured by RHC and the estimated echocardiographic PASP. The black line represents a hypothetical complete agreement between the two measurements

**Fig. 2:**
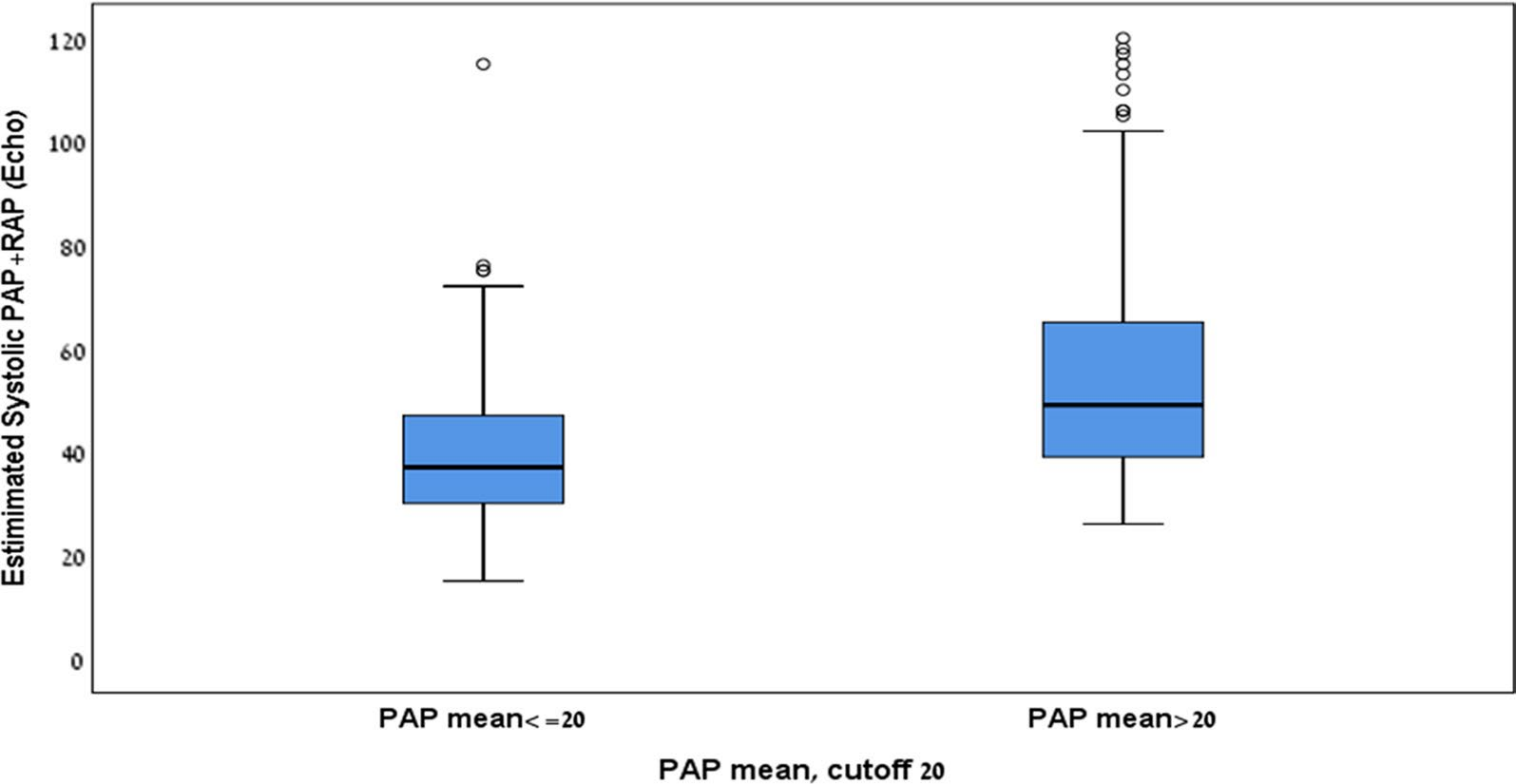
The estimated PASP per echocardiography in patients with confirmed diagnosis of PH (RHC mPAP > 20 mmHg) and in patients free of PH (RHC mPAP ≤ 20 mmHg)

**Fig. 3:**
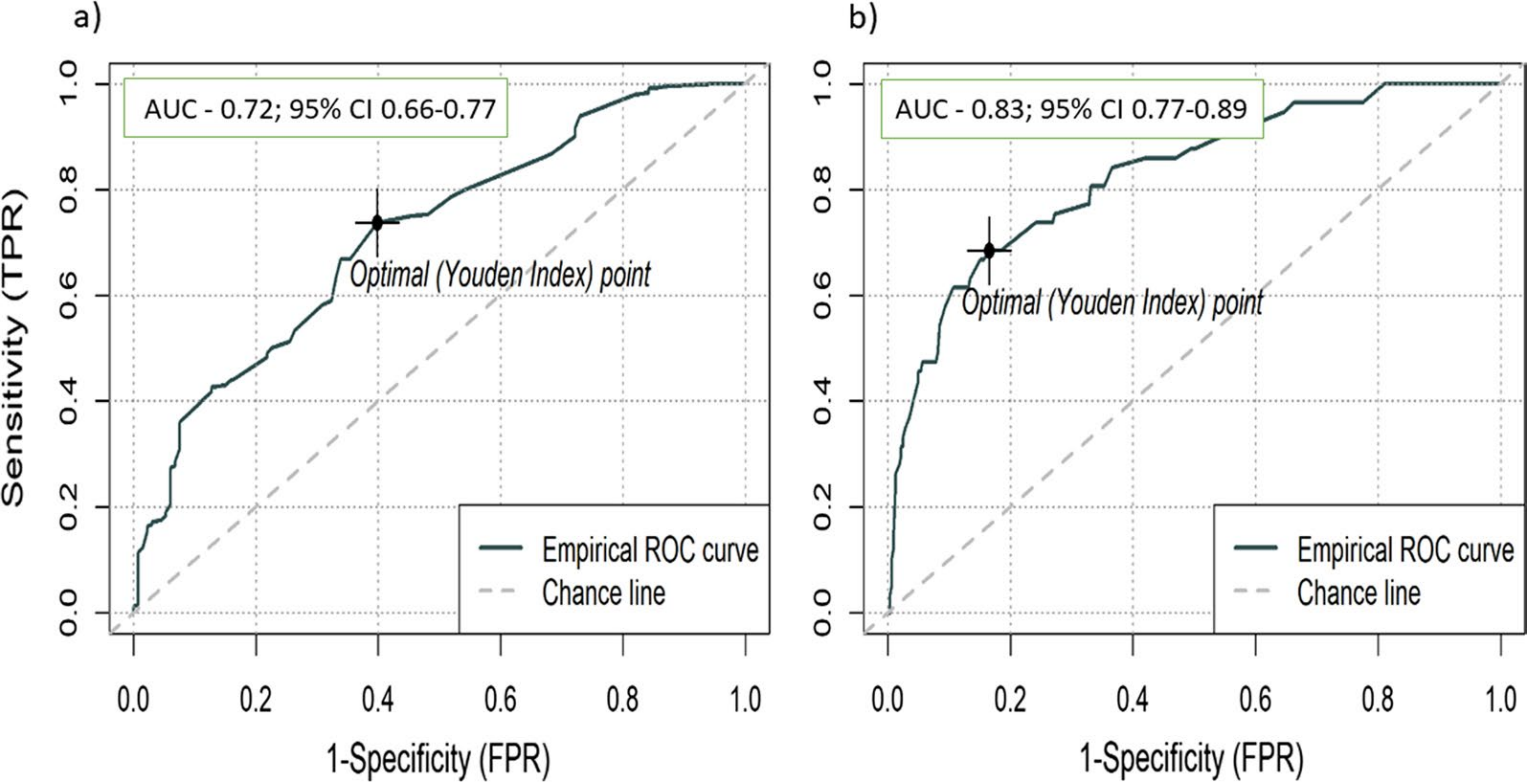
**a** A ROC curve demonstrating the prediction value of echocardiography to diagnose PH (mean PAP > 20 mmHg). AUC—0.72; 95% CI 0.66–0.77. Youden index—estimated PASP above 34 mmHg by echocardiography results in 0.73 sensitivity and 0.60 specificity. **b** A ROC curve demonstrating the prediction value of echocardiography to diagnose PH (mean PAP > 35 mmHg). AUC—0.83; 95% CI 0.77–0.89. Youden index—Estimated PASP above 52 mmHg by echocardiography results in 0.68 sensitivity and 0.83 specificity

When comparing the two methods for PASP estimation and measurement, we found the estimated PASP to be higher than that measured by RHC – estimated PASP of 49.5 ± 20.0 mmHg versus PASP absolute measurement of 42.5 ± 18.0 mmHg (*p* < 0.001). A moderate correlation between the two values was found according to Pearson’s correlation: r = 0.609, *p* < 0.01 (Fig. 1). The estimated PASP measured by echocardiography was significantly higher among those with confirmed diagnosis of PH (RHC mPAP > 20 mmHg) compared to those with pulmonary normo-tension; mean estimated PASP 54.6 ± 21.0 mmHg versus 40.5 ± 14.3 mmHg, *p* < 0.001 (Fig. 2). The prediction value of echocardiography to diagnose PH was assessed using ROC curve, with an area under the curve (AUC) of 0.72 (95% CI 0.66–0.77). The optimal echo derived PASP cutoff value to diagnose pulmonary hypertension was 34 mmHg (at the point of maximal Youden’s index) with sensitivity of 0.73 and specificity of 0.60 (Fig. 3a). When examining the prediction value to diagnose severely elevated pulmonary pressure (mPAP above 35 mmHg), the AUC is as high as 0.83 (95% CI 0.77–0.89). The optimal echo derived PASP cutoff value to diagnose severely elevated pulmonary pressure was 52 mmHg (at the point of maximal Youden’s index) with 0.68 sensitivity and 0.83 specificity (Fig. 3b).

**Fig. 4:**
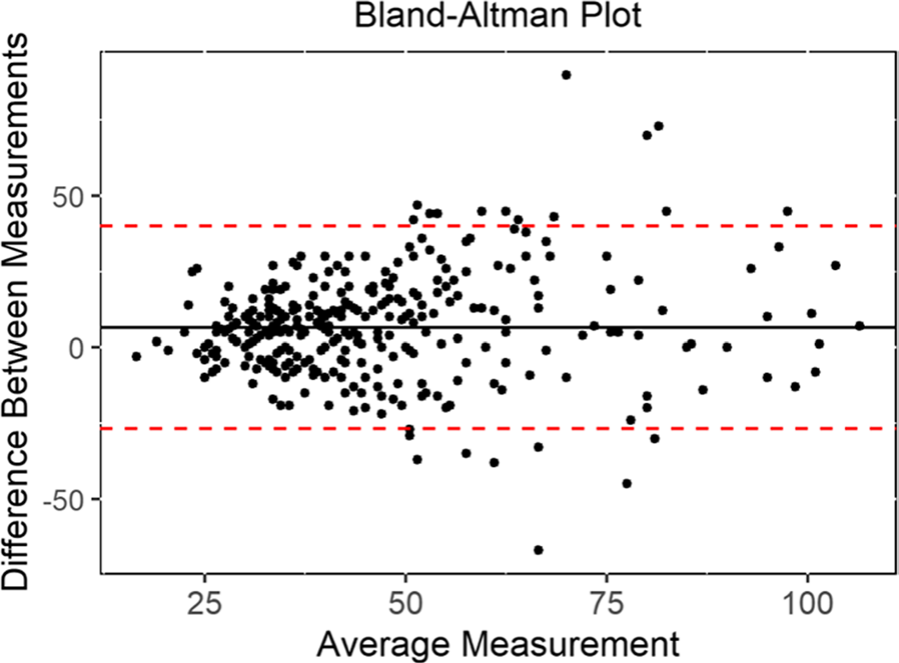
The Bland–Altman method was used to plot the difference in PASP measurements for each patient (RHC PASP measurement minus estimated PASP per echocardiogram) against the mean of the two measurements. The mean difference is 6.6 mmHg and the limits of agreement are − 26.9 and 40.1 mmHg (indicated by the broken lines)

The Bland and Altman method was used to describe the limits of agreement between the two measurement techniques. We have found the mean difference between estimated PASP and the measured PASP to be 6.6 mmHg, with an upper limit of 40.1 mmHg and lower limit of − 26.9 mmHg. Echocardiographic overestimation of PASP of more than 10 mmHg in 35.0% of the patients, and underestimation in 11.6% of the patients (Fig. 4) [21, 22].

Finally, in a separate analysis of the patients with no TR jet and no PASP estimation, we found that mPAP measured by RHC was not different compared to those with measurable TR jet—26.4 ± 11.5 mmHg versus 25.9 ± 11.5 mmHg. Factors associated with non-measurable TR jet were male gender (78.6% vs. 61.8%) and overweight (mean BMI 28.3 ± 4.8 vs. 25.93 ± 5.63) (Additional file 2: Baseline characteristics of patients with measurable TR compared to patients with no measurable TR).

## Discussion

PH is a well-known sequelae of advanced lung disease. Its diagnosis in LT candidates is crucial for the decision about the optimal medical and surgical treatment. Current guidelines rely on the echocardiographic assessment of PASP and do not recommend routine RHC for the diagnosis of PH. Previous studies assessing the correlation between PASP assessment by echocardiography and RHC measurements in patients with a variety of cardiopulmonary disorders found a good correlation between the two methods with an r value of 0.65–0.97 by Pearson’s correlation [15, 21, 23–32]. Other trials suggest that the echocardiographic measurement of pulmonary hypertension might be inaccurate, with high rates of either under or over estimation of PASP [11, 15, 33–35]. Moreover, some patients have no measurable tricuspid regurgitant jet, which makes the PASP estimation more challenging and often impossible [12].

Our study results confirms and expand upon previous observations with an updated analysis of a large group of LT candidates and accuracy analysis. Although the correlation between the echocardiographic estimation of PASP and the direct measurement of PASP by RHC was found to be moderate, the echocardiographic measurements were inaccurate and tended to overestimate the real PASP by more than 10 mmHg in ~ 1/3 of the patients, and to underestimate it in more than 1/10 of the patients. Results are consistent with previous publications in which echocardiography was found to overestimate PASP in 62.6% of the patients [34].

There are a few possible explanations for the limited correlation between the two diagnostic methods. First, mistakes may occur in accurate measurement of the peak tricuspid regurgitation signal. Suboptimal Doppler alignment or poor quality of the Doppler signals may cause overestimation and underestimation of the PASP. Moreover, when calculating trans tricuspid pressure gradients using the Bernoulli equation, the TR velocity is squared and multiplied by 4, meaning that small errors in the TR velocity measurements can result in major changes in the pressure estimation. Second, in order to estimate PASP, the pressure gradients across the tricuspid valve needs to be added to the estimate RAP, the latter derived from IVC dimeter and it's collapsibility. However, in many patients, IVC diameter is difficult to obtain, and even in those where measurement is possible, the accuracy between echo estimation of RAP and invasive measurement is as low as 34% with clear tendency toward echocardiographic overestimation [14, 36]. Third, the time lapse between the two studies may have contribute to the differences between modalities.

The limited accuracy of the echocardiographic measurements, and its moderate correlation with the gold standard RHC derived PASP, suggest that in the unique population of LT candidates, routine RHC may be mandatory in the pre-surgical evaluation, regardless of the echocardiographic estimation of PASP.

Moreover, in the group of patients with non-measurable TR jet and no echocardiographic estimation of PASP (10.9% of the candidates) the mPAP on RHC was similar to those with measurable TR, meaning that the absence of TR does not predict absence of PH. This might be explained by technically difficult echocardiographic studies in those patients who were found to be obese compared to the group of patients with measurable TR. Regardless from the reason, our results and others [37], suggest that invasive measurements are also inevitable in the pre-surgical evaluation of patients with non-measurable TR jet.

Since the prediction value of echocardiography PASP measurements improved significantly when using it to diagnose severely elevated pulmonary pressure, we suggest using this noninvasive method as a reliable tool for follow up and assessment of treatment response when PASP estimation exceeds 52 mmHg. Results are consistent with recent publication suggesting that the specificity and the positive predictive value of echocardiographic estimation of PASP increases with high cutoffs (38).

The study has a few limitations: First, it is based on a retrospective cohort. The echocardiographic interpretation and RHC measurements were not supervised or reviewed by a second physician and the two studies were not necessarily performed on the same day. Second, the echocardiographic estimation of PASP was based solely on the TR jet and other parameters used for the evaluation the right heart were not recorded. RHC related complications were not recorded as well. Nevertheless, to the best of our knowledge, this is the largest study of its kind which reflects the real-world data of LT candidates and their pre-surgical evaluation.

## Conclusion

In the pre-surgical evaluation of LT candidates, echocardiographic estimation of PASP had moderate correlation with the PASP measured by RHC, and tended to overestimate or underestimate RHC measurements. Echocardiography PSAP measurements were moderately discriminative to diagnose PH. The absence of TR did not preclude elevated PASP. Based on the study results, we recommend performing routine RHC to all LT candidates, regardless of the echocardiographic estimation of PASP or the presence of TR gradient.

## Supplementary Information

**Additional file 1:** Lung transplant etiologies.

**Additional file 2**: Baseline characteristics of patients with measurable TR compared to patients with no measurable TR.

## Data Availability

The data used and analyzed during the current study are available from the corresponding author on reasonable request.
