# Correction to: Preoperative evaluation of pulmonary hypertension in lung transplant candidates: echocardiography versus right heart catheterization

**DOI:** 10.1186/s12872-022-02771-x

**Published:** 2022-07-20

**Authors:** Tal Abu, Amos Levi, David Hasdai, Mordechai R. Kramer, Tamir Bental, Tali Bdolah‑Abram, Arthur Shiyovich, Abed Samara, Hana Vaknin‑Assa, Leor Perl, Dror Rosengarten, Yaron Shapira, Ran Kornowski, Keren Skalsky

**Affiliations:** 1 Faculty of Medicine, The Hebrew University of Jerusalem, Jerusalem, Israel; 2 Department of Cardiology, Rabin Medical Center – Beilinson Hospital, 39 Jabotinsky St., 4941492 Petach Tikva, Israel; 3 Rabin Medical Center, Pulmonary Institute, Petach‑Tikva, Israel; 4 Affiliated to Sackler Faculty of Medicine, Tel Aviv University, Tel Aviv, Israel

## Correction to: BMC Cardiovascular Disorders (2022) 22:53 10.1186/s12872-022-02495-y

Following the publication of the original article [1], the author name ‘Arthur Shiyovich’ has been misspelled as ‘Arthur Shyovich’

The original article has been corrected.
