# Supplementary material for: Preoperative evaluation of pulmonary hypertension in lung transplant candidates: echocardiography versus right heart catheterization
Source: BMC Cardiovasc Disord. 2022 Feb 16;22:53. doi: 10.1186/s12872-022-02495-y (PMC8851783; doi:10.1186/s12872-022-02495-y)
Supplement: Supplementary file 1 — Additional file 1: Lung transplant etiologies. [file 12872_2022_2495_MOESM1_ESM.pdf]

Supplementary Table 1 – Lung transplant etiologies

	All patients; N=393
ILD (%)	52.2
COPD (%)	30.5
PAH (%)	5.9
Bronchiectasis (%)	2.5
Other genetic conditions (%)	7.4
No record (%)	1.5

*COPD – Chronic obstructive disease, ILD – Interstitial lung disease, PAH –Pulmonary arterial hypertension.
