# Supplementary material for: Preoperative evaluation of pulmonary hypertension in lung transplant candidates: echocardiography versus right heart catheterization
Source: BMC Cardiovasc Disord. 2022 Feb 16;22:53. doi: 10.1186/s12872-022-02495-y (PMC8851783; doi:10.1186/s12872-022-02495-y)
Supplement: Supplementary file 2 — Additional file 2: Baseline characteristics of patients with measurable TR compared to patients with no measurable TR. [file 12872_2022_2495_MOESM2_ESM.docx]

**Supplementary Table 2 – Baseline characteristics of patients with measurable tricuspid regurgitation (TR) compared to patients with no measurable TR.**

|  | | **Measurable TR jet; N=351** | **No measurable TR jet;  N=42** | **P value** |
| --- | --- | --- | --- | --- |
| Age, mean (years) | | 61.6±8.4 | 60.4±7.5 | 0.40 |
| Male gender (%) | | 61.8 | 78.6 | 0.03 |
| Follow up, mean (months) | | 22.7±18.8 | 26.8±25.6 | 0.20 |
| COPD (%) | | 41.6 | 38.1 | 0.67 |
| Diabetes (%) | | 34.2 | 45.2 | 0.16 |
| Hypertension (%) | | 35.9 | 28.6 | 0.35 |
| Hyperlipidemia (%) | | 37.9 | 71.4 | 0.24 |
| Chronic kidney disease (%) | | 7.4 | 4.8 | 0.53 |
| Current smoking (%) | | 37.9 | 38.1 | 0.83 |
| Past smoking (%) | | 17.9 | 21.4 |  |
| BMI, mean | | 25.9±5.6 | 28.4±4.8 | 0.04 |
| CAD history (%) | | 22.8 | 33.3 | 0.21 |
| Echocardiography | Estimated EF, mean (%) | 59.1±5.2 | 58.6±6.0 | 0.53 |
|  | RV dysfunction (%) | 12.0 | 9.5 | 0.80 |
|  | PASP estimated by echocardiography, mean (mmHg) | 49.5±20.0 | NA* |  |
| RHC | RA pressure, mean (mmHg) | 5.1±4.7 | 5.2±4.4 | 0.89 |
|  | PAP, mean (mmHg) | 25.9±11.5 | 26.4±11.5 | 0.76 |
|  | PA sys, mean (mmHg) | 42.4±18.0 | 43.0±17.5 | 0.85 |
|  | PCWP, mean (mmHg) | 9.6±6.1 | 11.1±5.8 | 0.14 |
|  | PVR, mean (mmHg) | 4.2±3.4 | 4.1±4.0 | 0.87 |
|  | CO, mean (L/min) | 4.4±1.4 | 4.3±1.3 | 0.58 |
|  | CI, mean (L/min/m^2^) | 2.5±0.8 | 2.3±0.6 | 0.14 |

*BMI – Body mass index, CAD – Chronic artery disease, CI – Cardiac index, CO – Cardiac output, COPD – Chronic obstructive pulmonary disease, EF – Ejection fraction, NA – not applicable, PA – Pulmonary artery, PAP – Pulmonary arterial pressure, PASP – Pulmonary artery systolic pressure, PCWP – Pulmonary capillary wedge pressure, PVR – Pulmonary vascular resistance, RA – Right atrium, RHC – Right heart catheterization, RV – Right ventricle, TR – Tricuspid regurgitation.
